# Simulations of stressosome activation emphasize allosteric interactions between RsbR and RsbT

**DOI:** 10.1186/1752-0509-7-3

**Published:** 2013-01-15

**Authors:** Ulf W Liebal, Thomas Millat, Jon Marles-Wright, Richard J Lewis, Olaf Wolkenhauer

**Affiliations:** 1 Department of Systems Biology & Bioinformatics, Institute of Computer Science, University of Rostock, 18051, Rostock, Germany; 2 Institute for Cell and Molecular Biosciences, Faculty of Medical Sciences, Newcastle University, Newcastle-upon-Tyne, NE2 4HH, UK; 3 Institute of Structural and Molecular Biology, School of Biological Sciences, Edinburgh University, Edinburgh, EH9 3JR, UK; 4 Institute for Advanced Study (STIAS), Wallenberg Research Centre at Stellenbosch University, Stellenbosch, 7600, South Africa

**Keywords:** *Bacillus subtilis*, Stressosome, Signalling, Cellular automaton, Stress response

## Abstract

**Background:**

The stressosome is a bacterial signalling complex that responds to environmental changes by initiating a protein partner switching cascade, which leads to the release of the alternative sigma factor, σ^B^. Stress perception increases the phosphorylation of the stressosome sensor protein, RsbR, and the scaffold protein, RsbS, by the protein kinase, RsbT. Subsequent dissociation of RsbT from the stressosome activates the σ^B^ cascade. However, the sequence of physical events that occur in the stressosome during signal transduction is insufficiently understood.

**Results:**

Here, we use computational modelling to correlate the structure of the stressosome with the efficiency of the phosphorylation reactions that occur upon activation by stress. In our model, the phosphorylation of any stressosome protein is dependent upon its nearest neighbours and their phosphorylation status. We compare different hypotheses about stressosome activation and find that only the model representing the allosteric activation of the kinase RsbT, by phosphorylated RsbR, qualitatively reproduces the experimental data.

**Conclusions:**

Our simulations and the associated analysis of published data support the following hypotheses: (i) a simple Boolean model is capable of reproducing stressosome dynamics, (ii) different stressors induce identical stressosome activation patterns, and we also confirm that (i) phosphorylated RsbR activates RsbT, and (ii) the main purpose of RsbX is to dephosphorylate RsbS-P.

## Background

The stressosome signalling complex of *Bacillus subtilis* is activated in response to diverse environmental stresses, including ethanol, temperature, UV light, and osmolarity, and initiates a protein partner switching cascade that leads to the release of the alternative transcription factor σ^B^[1-3]. The complex is the most upstream component so far characterised of the environmental arm of the general stress response in *B. subtilis*[1,4]. Its activation results in the upregulation of nearly 200 genes, including proteins which provide protective adaption to environmental change [5-8].

**Figure 1:**
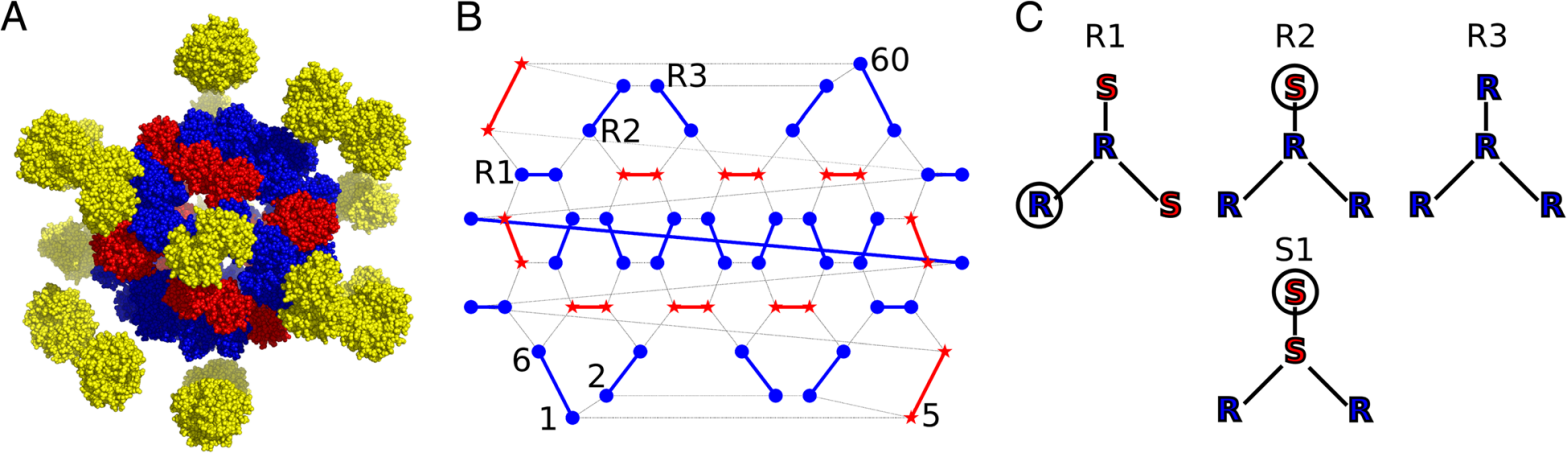
**Molecular composition of the stressosome. (A)** The atomic model of the stressosome [10] is coloured by domain; C-terminal, RsbR-STAS domain is blue, N-terminal RsbR domain is yellow, RsbS is red and RsbT is not shown for clarity. **(B)** The stressosome as a two-dimensional network, with RsbR monomers (blue circles) connected by blue lines to display the distribution of RsbR dimers. Similarly, RsbS monomers (red stars) are connected by red lines to form dimers. Close contact between neighbouring proteins is represented by dashed lines. The numbers indicate the scheme we use to identify individual proteins in the structure. Three different neighbourhood configurations for RsbR are exemplified with the R1, R2, and R3 tags. **(C)** The four different neighbourhoods in the stressosome structure will experience different protein cooperativity effects on the phosphorylation reaction and thus different phosphorylation rates of the central protein. In the description of the neighbourhood composition, we always start by naming the unpaired protein; these are circled in the figure.

The stressosome has a supra-molecular structure of a truncated icosahedron [9,10] and consists of the presumed sensor protein, RsbR, and the scaffold protein, RsbS [11-13]. The cryo-EM structure of the stressosome revealed its molecular organisation with 40 copies of RsbR associated with 20 RsbS molecules (arranged in homodimers) (Figure 1A) [10]. In the ground state, 20 RsbT molecules are bound by 20 copies of RsbS [10]; RsbT dissociates from the stressosome following activation by environmental stress [11]. Five paralogues of RsbR are also present in *B. subtilis*: RsbRA, -B, -C and -D (formerly RsbR, YkoB, YojH, YqhA) [13,14] all of which retain the ability to form functional stressosomes with RsbS [9,14,15]. The fifth paralogue, YtvA, mediates the stress response to blue-light [16,17] and is also capable of forming stressosome complexes, at least *in vitro* (Marles-Wright and Lewis, unpublished). This ability to form complexes appears to stem from the high sequence conservation of the common C-terminal, STAS domains possessed by these proteins [18]. By contrast, the N-terminal domains of the paralogues are highly variable, suggesting differences in either stress perception, or the interactions with RsbT [9,15].

**Figure 2:**
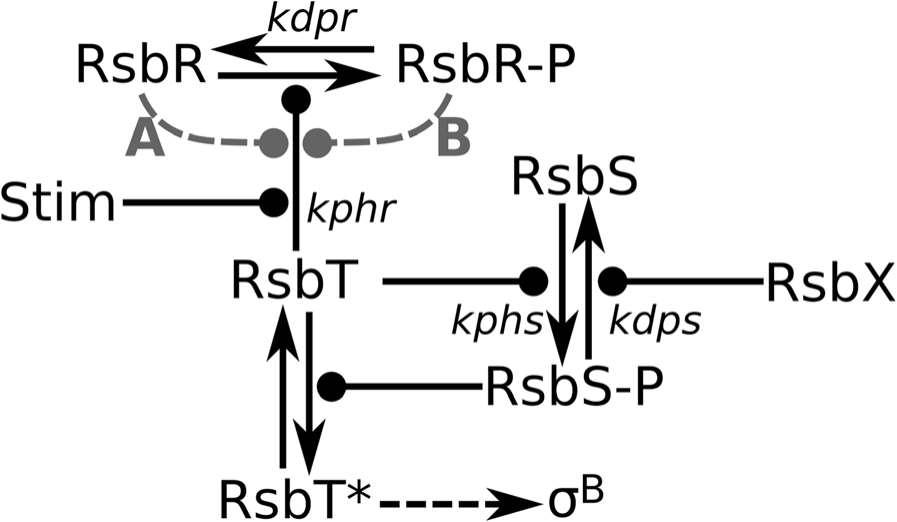
**Schematic of the reactions of the stressosome.** The reactions take place on the icosahedral stressosome structure, except for RsbT*, which is cytoplasmic RsbT. RsbT* initiates the general stress response by binding to RsbU and the subsequent release of σ^B^ through a partner switching mechanism. ‘Stim’ represents stimulation of the stressosome by a stressor. We tested three models of interactions between RsbR and RsbT: no interactions; RsbR as an activator of RsbT **(A)**; RsbR-P as an activator of RsbT **(B)**. The reaction parameters correspond to those of Table 1. Arrows represent reactions and lines with circles denote activation.

The role of the stressosome is the binding and the controlled release of RsbT in response to stress signals. Both RsbR and RsbS are necessary for the association of RsbT in the stressosome [14,19]. In stress-free conditions, a significant proportion of RsbR molecules are phosphorylated, whereas RsbS remains non-phosphorylated [20,21]. The imposition of stress leads to an increase in the phosphorylation levels of RsbR (Figure 2) [14,19-21], which is a requirement for the subsequent phosphorylation of RsbS by RsbT [19,22,23]. As the level of phosphorylated RsbS increases, the affinity of RsbT for the stressosome decreases (Figure 2) [20,24], resulting in the dissociation of RsbT. The released RsbT activates the protein phosphatase RsbU [24] and the activation of the partner switching cascade which ultimately leads to the release of σ^B^ from its quiescent complex with anti-sigma factor, RsbW [1-3]. Once released, σ^B^ directs RNA polymerase to the promoters of genes of the general stress regulon to stimulate their expression [11]. To reset this switch, the phosphorylation statuses of both RsbS and RsbR must be returned to pre-stress levels to allow RsbT to re-associate with the stressosome. The dephosphorylation of RsbS and/or RsbR appears to be catalysed by the phosphatase, RsbX [11,25]. The properties of the stimuli that activate the general stress response can be summarised in two categories, (i) environmental stress, and (ii) energy stress [26,27], both of which activate a phosphatase for RsbV-P [11,28]. Environmental stress (ethanol, UV-light, NaCl) is transmitted via the stressosome to activate the phosphatase RsbU whereas energy stress (glucose limitation) leads to the stressosme-independent activation of phosphatase RsbP [29]. However, there is insufficient knowledge of the phosphorylation dynamics of the stressosome because of the limitations of the experimental methods applied thus far. For instance, it is not known how the perception of an environmental signal causes the increase in RsbR and RsbS phosphorylation levels. Functional explanations for the existence and the mechanisms of the four RsbR paralogues are also missing; the paralogues have broad and overlapping sensitivities regarding stress stimuli [15].

Microbiological, molecular, and biochemical techniques have provided essential, general knowledge of the protein interactions and chemical reactions of the stressosome, but they are insufficient to understand the molecular events taking place in the complex. Here, we use computational modelling of the cryo-EM stressosome structure to test three hypotheses about the protein interactions within it, to gain insight on the spatial events associated with RsbR phosphorylation and their regulatory capacities. We compared three models: (i) ‘no cooperativity’, where the phosphorylation reactions in the stressosome are independent of neighbouring proteins; (ii) ‘substrate activation’, in which non-phosphorylated protein neighbours stimulate phosphorylation; and (iii) ‘product activation’, where phosphorylation is increased by the presence of phosphorylated neighbours. We evaluated the simulation results by comparing them with published data and found the ‘product activation’ model provided the best fit to the experimental data. A comparison of our simulation results with the signal-response data of Marles-Wright *et al.* (2008) [10] revealed identical sigmoidal stressosome activation patterns for salt and ethanol treatment, indicating that the activation dynamics of the stressosome are independent of any specific stressor.

## Results

### Stressosome fractional phosphorylations are comparable between experiment and simulation, though independent of the model type

**Figure 3:**
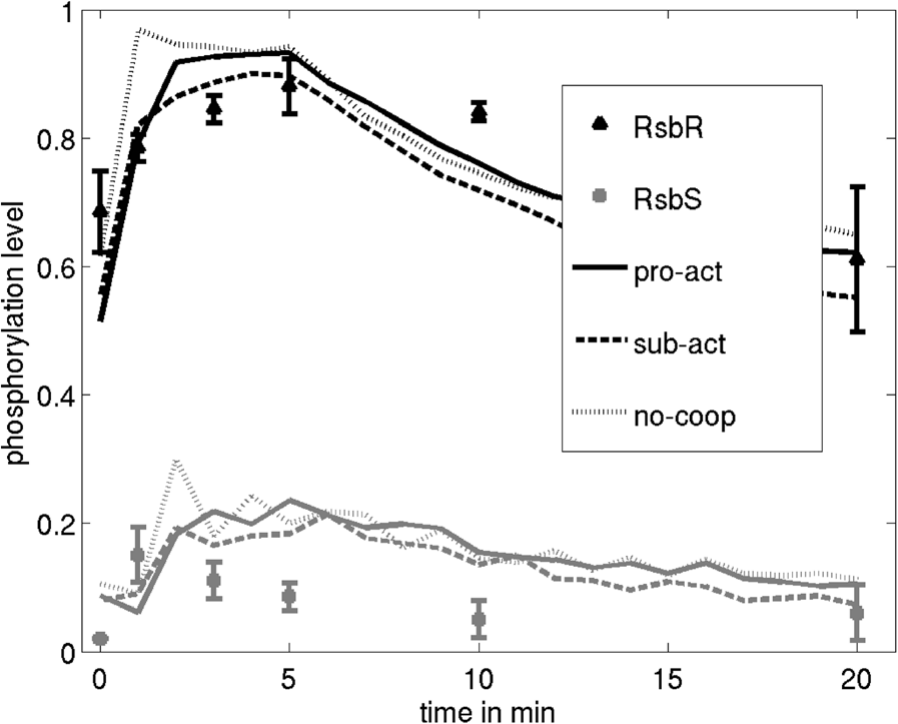
**Fractional phosphorylation of RsbR and RsbS during stress.** Comparison of mean and variance of RsbR (triangles) and RsbS (squares) phosphorylation levels during NaCl or ethanol induction and simulation results (lines). The experimental data were extracted from Kim *et al.* (2004) [20]. The three different models for simulation are: the product activation model (continuous line), the substrate activation model (dashed line), and the no-cooperativity model (dashed-dotted line). Stress is simulated assuming an increase in the phosphorylation probability of RsbR, *kphr*, to 1. In the simulation stress is stopped at 5 min by reversion of *kphr* to the according stress-free value of 0.1.

We compared our simulation results with the experimental data from Kim *et al.* (2004) [20] (similar results were reported by Eymann et al. [21]). The authors measured the fractional phosphorylation of stressosome components RsbR and RsbS during exposure to NaCl and ethanol [20]. The parameter settings used for fitting the observations are given in Table 1 and observations (markers) and simulations (lines) are shown in Figure 3. Activation of the stressosome is simulated with an increase in RsbR phosphorylation probability from 0.1 to 1. In the experiment, the fractional phosphorylation of RsbR decreased after 5 min, while levels for RsbS decreased after only 1 min. To simulate this apparent stress adaptation, we reset the RsbR phosphorylation probability from 1 to 0.1 after 5 minutes and therefore the stress is only active in the simulation between 0 and 5 minutes.

**Table 1:** Parameter values for the probabilities of reactions in the stressosome

**Parameter**	**Meaning**	**Value**
*kphr*	Phosphorylation of RsbR	0.1 / 1
*kdpr*	Dephosphorylation of RsbR-P	0.06
*kphs*	Phosphorylation of RsbS	0.4
*kdps*	Dephosphorylation of RsbS-P	1

The parameter *kphr*, the phosphorylation probability, has two values, the first for stress-free, the second for stress-response conditions. To consider the effects of neighbours, the parameter *kphr* is multiplied by an allosteric parameter (Table 3).

In the Kim *et al.* (2004) [20] study, the fractional phosphorylation of RsbR pre-stress is around 0.7, and is thus similar to our simulation results of 0.6. The peak phosphorylation levels are also comparable, although shifted to later times for RsbS in the simulation. In the experiments of Kim *et al.* (2004) [20], the RsbS fractional phosphorylation level increased rapidly following stress induction, whereas in our model RsbS phosphorylation increased only after the phosphorylation of RsbR. The RsbR fractional phosphorylation decayed faster in the simulation in comparison to the experiment, but both experiment and simulation arrived at comparable values of 0.6 towards their respective conclusions.

We did not attempt to model the long-term regulation of stressosome activation because that requires the additional consideration of changes in the σ^B^ operon gene expression levels. Therefore, while we captured the ranges of RsbS and RsbR phosphorylation, the dynamics of the RsbS deactivation process are not reproducible in our framework. A notable difference between the models was the faster activation of RsbR and RsbS in the ‘no-cooperation’ model in Figure 3. This faster response is caused by an increase in the phosphorylation probability for all RsbR molecules, because, unlike in the other models, the phosphorylation probability is not restricted to neighbouring molecules. All three models perform comparably in relation to the activation profiles in Kim *et al.* (2004) [20], indicating that another parameter must determine the biological significance of one model over the others.

### The stressosome acts through a product activation model based on the sigmoidal signal-response characteristics seen *in vivo*

**Figure 4:**
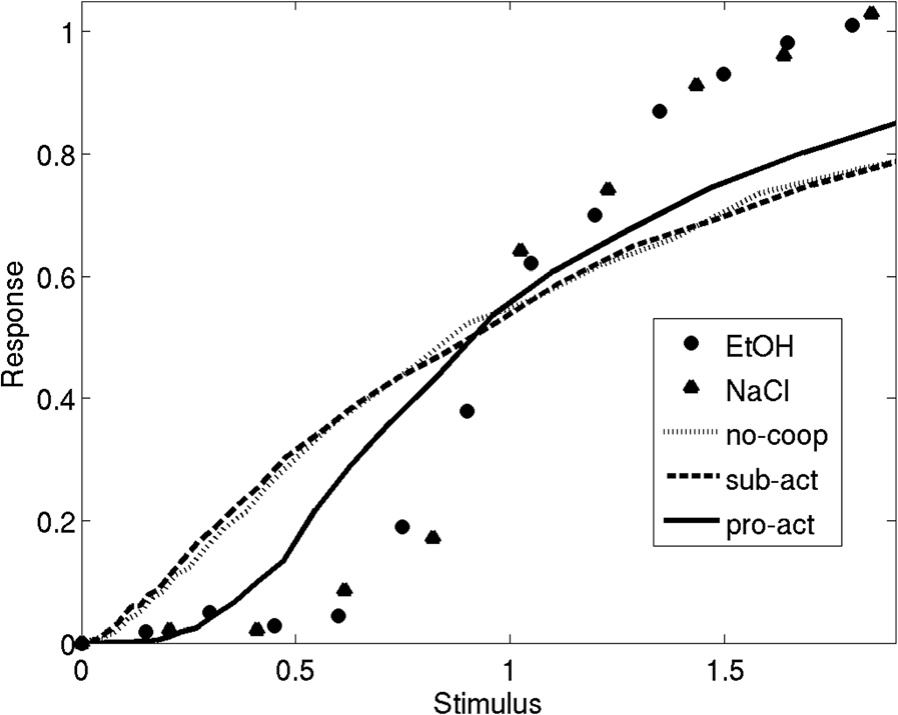
**Stimulus–response characteristics of the stressosome.** The different stimuli used in experiments by Marles-Wright *et al.* (2008) [10] were ethanol (triangles) and NaCl (circles). The simulations according to the three models tested are: product activation (continuous line), substrate activation (dashed line), no cooperation (dashed-dotted line). As experiment and simulation use different stimuli (NaCl, ethanol and *kphr*, respectively), and response definitions (β-galactosidase and RsbS phosphorylation) the stimuli and responses were normalised according to Equation 1. Ethanol and NaCl activate the stressosome in an identical manner, leading to identical stimulus–response characteristics. Only the product activation model approximates the experimentally observed sigmoidal character of this response. The parameters are identical to the reproduction of the Kim *et al.* (2004) [20] data and are shown in Tables 1 and 3.

To determine the crucial parameter that separates the three models, and to understand the phosphorylation dynamics of the structure of the stressosome, stress activation was modelled as a function of the increase in RsbR phosphorylation probability. Marles-Wright *et al.* (2008) [10] measured the β-galactosidase activity using a σ^B^ dependent *lacZ* reporter in response to different concentrations of the stressors NaCl and ethanol. A sigmoidal signal-response curve for both these environmental stressors was observed [10]. The sigmoidal signal-response was not observed during the stressosome-independent activation of σ^B^ by energy stress, suggesting strongly that the sigmoidal environmental stress response is stressosome-specific. We evaluated our simulation using these data because the direct outcome of the simulation is the RsbS fractional phosphorylation, which correlates directly to the release of RsbT from the stressosome and to the activation of σ^B^. To compare experiments and simulations, the experimental data were normalised as described in the Methods section. Strikingly, the experimental data for the stressosome response generated for ethanol (triangles) and NaCl (squares) coincide almost perfectly after normalisation (Figure 4). Consequently, the stressosome response is identical for these two different signals. Among the three models generated, only the ‘product-activation’ model resulted in a signal-response curve with a comparable sigmoidal character (pro-act curve in Figure 4), where the deviations from the experimental data are probably rooted in the model simplifications.

### The stressosome model captures RsbX titration experiments if RsbS is the only target of RsbX

**Figure 5:**
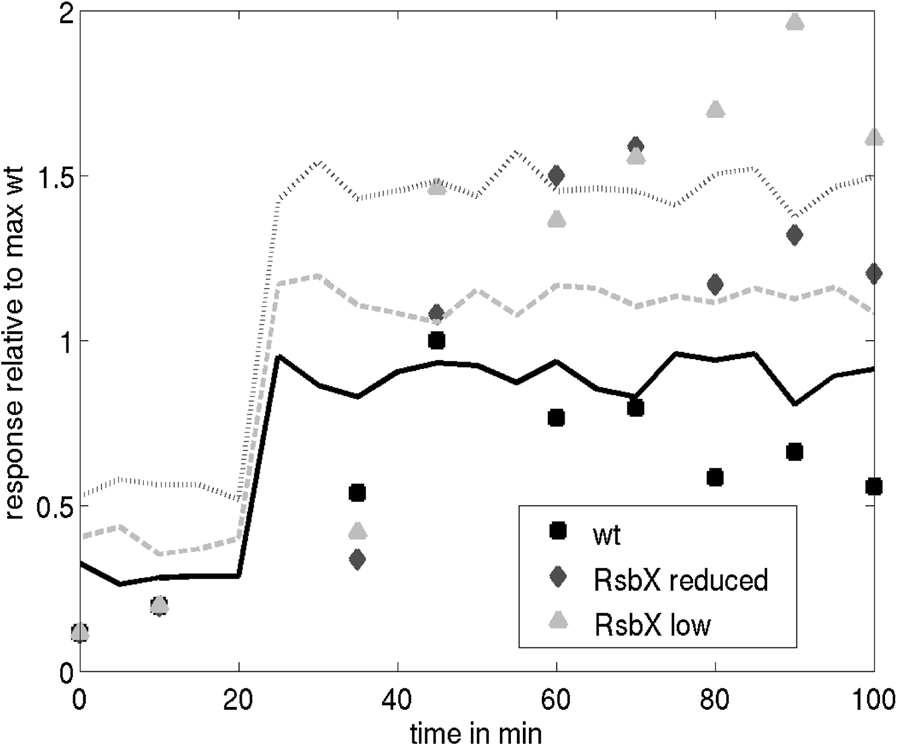
**Effect of reduced levels of RsbX on stress activation of σ**^**B**^**.** Three data-sets from Völker *et al.* (1997) [25] were digitized in which the level of RsbX is controlled by the addition of IPTG (BSA46 [wild type], squares; BSA337+1 mM IPTG [RsbX reduced], diamonds; BSA337+0.1 mM IPTG [RsbX low], triangles). The simulation was performed with the ‘product activation’ model. The responses of experiment and simulation are normalised to the maximum response of the wild type. The lines represent simulations with parameters as listed in Tables 1 and 3 but with appropriately adapted dephosphorylation probabilities (*kdps*), wt with *kdps*=1 (continuous line); reduced RsbX with *kdps*=0.6 (dashed line); low RsbX with kdps=0.1 (dashed-dotted line). The activation of the stressosome by ethanol (experiment) or by increase in *kphr* (simulation) both took place at 20 min.

We also evaluated the product activation model using experimental data from Völker *et al.* (1997) [25]. Here, the cellular concentration of the phosphatase RsbX was controlled by cloning it downstream of an IPTG inducible promoter. The ethanol stress response was tested by titrating the cellular levels of RsbX with IPTG. Yet again, the experimental outputs were measured using a σ^B^ dependent β-galactosidase reporter gene fusion, whereas the simulations produced fractional phosphorylation levels of RsbS. As described above, these two measures correlate directly because RsbS phosphorylation leads directly to σ^B^ activation. We normalised the two data sets internally with their highest unperturbed output, *i.e.* wild type β-galactosidase activity and RsbS phosphorylation. Stress was applied at 20 min by the addition of ethanol in the experiment and by increasing the phosphorylation parameter of RsbR, *kphr*, from 0.1 to 1 in the simulation. Since RsbX is a phosphatase we altered the values of probability of dephosphorylation of RsbS, *kdps*, and RsbR, *kdpr*, in our model. We reproduced (Figure 5) the three data sets by Völker *et al.* (1997) [25] using three values for *kdps*: 1 (wild type, continuous line), 0.6 (RsbX reduced, dashed line), and 0.3 (RsbX low, dash-dotted line). In the simulation, a reduction in the dephosphorylation of RsbR failed to reproduce the experimental data, because the response after activation remained constant at the level of the wild type response (not shown). The response in our simulations was faster than the experimental data, because we used RsbS phosphorylation as the activity proxy, and thus omitted the additional time delay caused by the expression of the reporter gene. The time delay between maximum RsbS phosphorylation and maximum reporter protein signal is about 15 to 25 minutes [20], which only slightly smaller compared to the approximate 30 minutes delay of simulation and measurements in Figure 5. The experimental results of Völker *et al.* (1997) [25] are thus explained in the model by assuming that the stressosome and thus the environmental stress response is reset by the unique dephosphorylation of RsbS-P by RsbX.

### The computational model is able to reproduce a variety of experimentally determined stressosome phenotypes

By relating experimentally measured σ^B^ activities to the fractional phosphorylation of RsbS (the model output), we were able to compare the simulations with a number of published experiments (see Table 2). First, Akbar *et al.* (2001) [13] measured stress responses for strains with mutations in both RsbRA and RsbRB, and in either RsbRC or RsbRD, or both (see Table 2). The major outcome is that RsbRC and RsbRD increase in pre-stimulus and post-stimulus β-galactosidase levels. In the experiments of Martinez *et al.* (2010) [30], stress was induced by the transition to stationary phase (energy stress), and it was thus reported by these authors that RsbRC and RsbRD can sense energy stress. Our unbiased simulations support this notion and provide clues about the kinetic implications of these findings. The most direct way to replicate this finding in the simulation is to increase the phosphorylation likelihood for RsbS, *kphs*. Thus, RsbRC and RsbRD are more efficient than RsbRA and RsbRB in inducing the RsbT kinase activity towards RsbS during energy stress stimulation. The mixture of RsbRC and RsbRD, with stress-insensitive RsbRA and RsbRB, lowers the apparent activation of RsbS (Additional file 1 Figure S1) [30]. Second, Kim *et al.* (2004) [14] detected a hyperbolic β-galactosidase stress response for RsbRD instead of the sigmoidal response seen for RsbRA and RsbRB. As shown in Figure 4, the models of ‘substrate activation’ and ‘no cooperation’ produce hyperbolic responses while the sigmoidal response generated by the ‘product activation’ model is caused by the allosteric behaviour of RsbR interactions (see Table 3). Although the data of Kim *et al*. (2004) [14] are in the form of a time course and the sigmoidal property is derived from a dose–response curve, a time course can be controlled by the dose–response if the stressosome adapts faster to the stimulus than the stimulus changes itself. On the basis of this assumption, the model predicts that RsbRD is less allosteric than RsbRA. Finally, the cellular automaton enables qualitative analysis of RsbR mutations. Amino-acid substitutions on certain positions of RsbRA result either in elevated or reduced pre-stress output while maintaining a wild type stress response (Table 2) [31,32]. Since the stress response is unaffected, the protein interactions of the stressosome are not involved and thus the truth table remains unchanged. Based on the assumption that stress stimulation increases RsbR phosphorylation by RsbT, the substitutions either activate or inhibit RsbT without stress stimulation if the mutations increase or decrease the pre-stimulus response, respectively. However, the stimulation of RsbT after stress perception proceeds undisturbed.

**Table 2:** Comparison of experimental observations with simulation

**Experiment**	**Phenotype**	**Reference**	**Model adaptation**	**Simulation**
reduced RsbX	increase in β-Gal response	[25]	decrease in *kdps*	increase in post-stress RsbS-P
ΔRsbR(AB), ΔRsbR(ABC), ΔRsbR(ABD)	alteration in pre- and post-stress β-Gal response	[13]	increase in *kphs*	increase in RsbS-P
ΔRsbR(ABC), RsbRD stressosome	hyperbolic response	[14]	increase in background phosphorylation (allost. par.) decrease of cooperativity	hyperbolic response
RsbR, RsbS phosphorylation after stimulus	transient increase in phosphorylation level	[20]	increase in *kphr*	increase of RsbR-P
stimulation with different stress level	sigmoidal dose–response curve	[10]	adaptation of the allosteric parameter for R1 and R2 neighbours	allosteric activation of RsbT by RsbR-P
RsbRA T86A, N129A, Q142A, etc.	elevated basal β-Gal level but wild type stress response	[31,32]	increase of pre-stress *kphr*	increase of pre-stress RsbR-P
RsbRA L141A, Q147A, L149R	reduced basal β-Gal level but wild type stress response	[32]	decrease of pre-stress *kphr*	decrease of pre-stress RsbR-P

The phosphorylation of RsbS is correlated to the β-galactosidase response because phosphorylated RsbS releases RsbT, the activator of σ^B^.

**Table 3:** Allosteric parameters for the different models

**Triangle**	**000**	**010**	**011**	**100**	**110**	**111**
General						
R3	0.7	0.7	0.7	={010}	={011}	0.7
S1	0	0	1	0	0	0
No cooperation						
R1	1	1	1	1	1	1
R2	1	1	1	1	1	1
Substrate activation						
R1	0.7	0.7	0	0.5	0.5	0
R2	1	0.7	0.5	0	0	0
Product activation						
R1	0	1	0	1	1	0
R2	0	1	1	0	0	0

The first column represents the neighbourhood composition (compare to Figure 1) and columns two to six represent different phosphorylation states of the models and their allosteric parameter. The section labelled ‘General’ contains the model independent allosteric parameters, whereas the other three sections show the parameters for their respective models.

## Discussion

Herein, we present the first computational model of the stressosome based on a Boolean representation of phosphorylation. The consequences of the unique neighbourhood compositions in a truncated icosahedron were simulated in a cellular automaton-like computational environment wherein the future state of a protein is based upon the phosphorylation status of its neighbouring proteins. We analysed simulated time course data of RsbR and RsbS phosphorylation, as well as steady state phosphorylations at different stress inputs and compared them to data from the literature. For simplicity, we disregarded any effects that may originate from the interactions between dimers of RsbR and RsbS as there are no experimental data available on such effects. Moreover, we also ignored the potential for the RsbR paralogues to display a localisation bias within the stressosome (e.g. the R3 neighbourhood). This is, because, to date, there is no information available on the localisation patterns of RsbR and its paralogues within the stressosome. Although four RsbR paralogues contain two threonine residues as potential phosphorylation sites [22], our model considers only single phosphorylations of RsbR. The double phosphorylation of RsbR occurs only in response to the imposition of strong stresses and the double RsbR phosphorylation actually limits stressosome activation [21]. We have avoided the double phosphorylation phenomenon in order to keep the model simple, whilst maintaining a model that is applicable for all but the most extreme of stressful incidents. The fitting of the model to the experimental data required a high phosphorylation status for RsbR molecules in a neighbourhood with only RsbR neighbours (R3-neighbourhood). A single phosphorylation of RsbRA (at T171) was measured in response to low and to moderate stress [21], whereas strong, growth inhibiting stress required double phosphorylation (T171 and T205). The phosphorylation of RsbRA at both sites is likely to attenuate σ^B^ activation and is not involved in the initial response to stress [21]. We considered only one phosphorylation event, as we sought to reproduce experiments that induced moderate stress levels (e.g. 4% ethanol), which do not require the double phosphorylation of RsbRA.

### Predictability of the stressosome cellular automaton

Whereas the majority of bacterial sensory systems consist of monomers or dimers, some systems, including the stressosome and chemotaxis arrays, form large complexes. Amongst the possible reasons for this phenomenon is an increase of the regulatory space; a sensor that interacts with its neighbours expands its input signal range. An adaptation of the interactions can thus affect the response. In terms of cellular automata, these interactions correspond to the update tables. What is the knowledge we can expect from such an abstraction? A cellular automaton is first and foremost a spatial model; it reproduces patterns like the distribution of black and white squares on a lattice. A different update table yields a different pattern, fitting an observation better, or worse. The best test of the stressosome cellular automaton is the direct observation of phosphorylation patterns. For example, our model predicts hyper-phosphorylation of RsbR in the neighbourhood of R3. This, however, is technically impossible to measure and probably biologically irrelevant. The purpose of the stressosome is the release of RsbT from phosphorylated RsbS, and thus probably no particular pattern but the total phosphorylation matters. Our cellular automaton allows the examination of different interactions by adapting the truth table in Table 3, and the effect of external inputs, as represented by the parameters of phosphorylation and dephosphorylation. In this context, predictions are hard to formulate because the output of an altered RsbS phosphorylation can be reproduced by a number of patterns generated by different update tables and input parameters. Consequently, we evaluated our model on existing data that enabled us to associate model parameters with biological functions.

### Phosphorylated RsbR cooperatively activates RsbT

The phosphorylation of RsbR is a requirement for the activation of the stressosome, because inhibition of the threonine residue targeted for phosphorylation in RsbRA (T171A) blocks stress response [14]. Furthermore, the phosphorylated form of RsbR was found to stimulate the kinase activity of RsbT [19]. Our simulations of the allosteric activation of RsbT by phosphorylated RsbR reproduced most successfully the published data on stressosome activation. This RsbR phosphorylation requirement also explains why RsbR is phosphorylated to high levels in stress-free conditions [20,21].

### Different stress signals induce constant increases in RsbT activity

Environmental stresses lead to an increase in RsbT kinase activity against RsbR and RsbS, either by direct interactions of RsbT with RsbR paralogues, or through some, as yet, undetermined secondary interaction [22]. Reanalysis of the data presented in Marles-Wright *et al.* (2008) [10] shows that the levels of the activation of RsbT in response to stress is independent of the nature of the stress (Figure 4). How is this achieved? The N-terminal domains of RsbR, presumed sensors (inferred in part because this domain of YtvA is a blue light sensor), may interact with a secondary messenger molecule, or with a protein that integrates the initial stress signal. A candidate for this possibility is Obg, a ribosome-interacting protein with unclear roles in sporulation and σ^B^ activation [33]. Ethanol and NaCl have similar physiological effects by inducing secondary oxidative stress (reactive oxygen species) in the electron transport chain [34], potentially linking these stressors with the stressosome. Whether RsbT activation requires the involvement of a small molecule, or a protein integrator, are aspects of the stress response that remain to be determined experimentally.

### RsbX only dephosphorylates RsbS during low and moderate stress events

*In vitro*, RsbX can dephosphorylate RsbS-P and RsbR-P, but the latter only at residue T205 [23]. The dephosphorylation reactions have also been studied *in vivo*[21], and the two approaches provide broadly consistent results. The inefficient dephosphorylation of RsbR T171-P by RsbX probably explains the slow decrease in RsbR phosphorylation observed by Kim *et al.* (2004) [20] (summarised in Figure 3), whereas RsbS was dephosphorylated rapidly. In our simulations we found a dephosphorylation probability for RsbR that is 16-fold lower than that for the dephosphorylation of RsbS-P (0.06 and 1, respectively, see Table 1). The stress response of strains expressing different levels of RsbX following a challenge with 4% ethanol has been tested [25] and such a challenge should lead to only a single phosphorylation in RsbRA at residue T171 [21]. Indeed, the data of Völker *et al.* (1997) [25] could only be reproduced in our model by assuming that RsbX was active as a phosphatase solely towards RsbS-P. A functional stressosome also requires a balanced phosphorylation status of RsbR. Experiments and simulation do not support the prior assumption that RsbX mediated the dephosphorylation of RsbR-P, though it is still formally possible at a low, but significant level.

## Conclusions

In reproducing numerous published experiments, our stressosome simulations add weight to a model in which RsbT is activated allosterically by phosphorylated RsbR. Our model also suggests that RsbX is only required to dephosphorylate RsbS to reset the stressosome to a pre-stress state. Furthermore, the normalization of the data of Marles-Wright *et al.* (2008) [10] shows that stressosome activation and thus phosphorylation dynamics are identical for different stressors. Our model forms the foundation for future computational experiments to explore the effects of phenomena for which the mechanism of their action is currently unknown. These experiments could explore the impact of RsbR T205 phosphorylation on stressosome activation, the impact on localisation constraints of RsbR paralogues in the stressosome, or the negative feedback exerted on the system via σ^B^ mediated control of RsbX expression. Our initial model provides a proof of the utility of using Boolean network simulations to model stressosome activation, as demonstrated by our modelling of the activation dynamics of the stressosome for moderate stresses. For a complex and fascinating molecule like the stressosome, many questions remain to be answered despite two decades of intensive research on the regulation of σ^B^. The limitations of biological experimentations in this system can be overcome by computational modelling, which is proving to be a valuable tool to shed light on the function of not only this system [35-37], but other signalling networks too [38]. Consequently, the application of cellular automata is likely to provide insight to other, highly symmetric molecules that are poorly understood, for instance, the co-ordinated assembly and disassembly of bacteriophage, viruses, and bacterial micro-compartments, the communication of enzymatic active centres in pyruvate dehydrogenase complex [39] and the dynamic effects of pore opening and closing on iron uptake in ferritins [40,41].

## Methods

### Geometric properties of the stressosome

The experimental information used to construct our models, including a description of the geometric properties that may affect allosteric behaviour, is summarised by the following. The basic units of the stressosome are twenty dimers of RsbR and ten dimers of RsbS. Each protein interacts with a homodimer partner, but the icosahedral structure requires two additional interaction partners for each protein. The stressosome structure is constructed in such a way that while RsbR homodimers can interact with each other, RsbS homodimers never directly interact with each other. These rules, along with the observed stoichiometry of the complex, yield a single, unique assembly (Figure 1A). The truncated icosahedron of the stressosome core can be visualized as a two-dimensional network, as in Figure 1B. Each protein is in the centre of a triangle whose corners are defined by its neighbouring proteins (Figure 1C). Because the edges in a geometric icosahedron are all equidistant, we adopted the simplifying assumption that all positions in the neighbourhood have the same interaction strength with the central protein. We then numbered the proteins of the icosahedral network representation, starting from ‘1’ in the lower left and finishing with ‘60’ at the top-right (Figure 1B). A second list associated each protein with its interaction partners, e.g. protein ‘1’ (RsbR) is neighboured by {‘5’, ‘2’, ‘6’}, (RsbS, RsbR, RsbR – we start enumerations with the solitary protein type, the circled protein neighbour in Figure 1C). If a protein is phosphorylated then a ‘1’ is assigned to it, otherwise its state is ‘0’.

### Representation of reactions

There is no experimental evidence about the effect of the stressosome phosphorylation status on the dephosphorylation rate and consequently we assumed that the dephosphorylation rates are constant and are not affected by the state of neighbouring proteins. Therefore, the transition from state ‘1’ to ‘0’ (equivalent to RsbR-P/RsbS-P dephosphorylation) in our model takes place with a predefined probability identical for each of the three models and which is independent of any neighbours. By contrast, we modelled the transition from state ‘0’ to ‘1’ (RsbR/RsbS phosphorylation by RsbT) to be dependent upon the phosphorylation status of neighbouring proteins (Table 3), consistent with the biochemical data of Chen *et al.* 2003 [19]. The phosphorylation probability is determined based on a pre-defined maximum phosphorylation probability, *kphr*. The value is chosen to best reproduce the phosphorylation magnitude and time-scale for experimentally measured data on the stress response (Table 1) [20,21].

In the stressosome, four different neighbourhood configurations (triangles) exist, which are summarised in Figure 1C. Of the four combinations, three place RsbR in the centre, and one places RsbS in the middle. Each neighbourhood has a different number of RsbT molecules associated with it and thus the activation of RsbT by RsbR and RsbS within these regions is presumed to differ. To account for this triangle-specific activation, we have introduced the ‘allosteric parameter’ (*p*_*a*_), which represents the ability of a triangle to stimulate RsbT to maximum activity. The allosteric parameter can take any value between 0 and 1, and is multiplied by the maximum phosphorylation probability. In addition, the phosphorylation state of the three neighbours affects RsbT activity in each triangle. By permutation, there are thus 22 possible phosphorylation states for the four triangles: three triangles have six phosphorylation states (see R1, R2, and S1 in Table 3) and one triangle has four phosphorylation states (see R3 in Table 3). The resulting 22 free allosteric parameters represent a challenge for reasonable quantification, and we have thus used biological insight to reduce their number.

### Effect of protein interactions on phosphorylation

An increase in RsbS phosphorylation has been measured as a function of increased levels of RsbR phosphorylation [19,23]. Therefore, the kinase activity for the triangle with RsbS in its centre (S1) is at maximum if all RsbR neighbours are phosphorylated. Moreover, neighbouring RsbS molecules must be non-phosphorylated because otherwise the kinase dissociates. Hence only S1 with neighbourhood {0,1,1} has an allosteric parameter of 1, all other five states are inactive (*p*_*a*_=0).

RsbR with three RsbR neighbours (R3) lacks a nearby RsbT kinase, because in the structure of the stressosome RsbT is always immediately adjacent to RsbS [10]. A value for the allosteric parameter of 0.7 for all models allowed the optimal reproduction of the data of Kim *et al.* (2004a) and of Marles-Wright *et al.* (2008) [10]. The phosphorylation of RsbR in R3 is independent of the status of the neighbours because it is isolated from direct phosphorylation by RsbT due to its neighbourhood composition, and the influence of its neighbours on its phosphorylation is therefore minimal. Two triangle combinations with a central RsbR remain: R1 with arrangement (RsbR, RsbS, RsbS) and R2, arranged (RsbS, RsbR, RsbR) (Figure 1C). The neighbourhood R2 has six different phosphorylation combinations: either none, one, or both of the two RsbR molecules are phosphorylated. These three states can occur in combination with phosphorylated and non-phosphorylated RsbS, but the central RsbR cannot be phosphorylated if the neighbouring RsbS is already phosphorylated, because the cognate RsbT would have dissociated. Similarly, R1 has six phosphorylation combinations and we show in the next section how we use the phosphorylation combinations to model different hypotheses of protein interactions in the stressosome.

### Model definitions

We developed three computational models to test their capacity to reproduce experimental data, and they differ in the way that RsbR activates the RsbT. The possible circumstances are that RsbR (i) activates, (ii) inhibits or (iii) has no effect on RsbT. In the model, an inhibiting effect of RsbR is indistinguishable from an activation of RsbR-P, therefore we investigated activation of RsbT by RsbR-P instead. The interactions of RsbR and RsbT are reflected in different allosteric parameter values for phosphorylation in the triangles R1 and R2. In the ‘no cooperation’ model we assumed that RsbT activation is independent of its neighbours, which corresponds in our framework to setting to 1 (constant maximum kinase activity) all the allosteric parameters in the triangle configurations (Table 3). In the ‘substrate activation’ model, non-phosphorylated RsbR stimulated RsbT and the allosteric parameter values increased from 0 to 1 with a decrease in the phosphorylation of RsbR. By contrast, the allosteric parameter increased from 0 to 1 along with an increase in the number of phosphorylated RsbR neighbours for the ‘product activation’ model. The specific values for the allosteric parameters were optimized empirically for the best reproduction of experimental data (Table 3).

### Boolean model simulations

The stressosome reactions were split into regular steps for the following rationale. First, we sought to compare two time periods, the time between two reactions of a given protein, referred to as the ‘waiting-time’, and the time during which all proteins in the stressosome react once, referred to as the ‘process-time’. If the process-time is smaller than the waiting-time, then a step-wise update rule is appropriate to approximate stressosome dynamics because the system appears step-wise regarding the waiting-time. Long waiting-times are a central assumption of the stochastic simulation algorithm used to simulate stochastic systems with low copy numbers comparable to the 60 proteins of a stressosome [42]. Second, a longer waiting-time than process-time for the stressosome is valid because after phosphorylation, the kinase has to exchange ADP for ATP in its active site and the phosphatase has to diffuse to the stressosome complex to catalyse its dephosphorylation.

In the simulation the initial phosphorylation state of RsbR and RsbS was randomly assigned with a probability of 50% for each to allow rapid equilibration of the system. The equilibrium was independent of the exact initial state which affects the relaxation time only. During a simulation step we determined for all 60 proteins, in random order, whether a phosphorylation reaction would occur or not. For instance, the triangle R2 has neighbours (RsbS, RsbR, RsbR) with a phosphorylation status {0,1,1} and the central RsbR is non-phosphorylated. From Table 3, it follows that the allosteric parameter for the ‘no-cooperation’ model is *p*_*a*_=1, for ‘substrate activation’ *p*_*a*_=0.5, and ‘product activation’ *p*_*a*_=1. To calculate the reaction probability, the allosteric parameter was multiplied by the maximum phosphorylation probability, *kphr*, which is 0.1 for stress-free and 1 for stressful conditions. Whether a reaction actually occurs was determined using a Monte-Carlo approach: the reaction probability was compared with a number drawn from a uniform distribution in the interval [0,1]. Only if the phosphorylation probability was smaller than the random number was phosphorylation deemed to have occurred. Dephosphorylation was determined similarly using the dephosphorylation parameter. Simulations were repeated 50 times while assuring that statistical properties did not change significantly. The model was implemented in Matlab(R) (7.11.0) and is available as Additional file 2: ‘Liebal_stressosome-matlab-model.zip’ for this article.

### Normalisation of signal-response data

The experimental data by Marles-Wright *et al.* (2008) [10] and our simulation results differ in their input and read-out variables and therefore, for comparison, they were normalised. The experimental data followed a sigmoidal shape and we used a hyperbolic tangent to characterise it:

(1) $$f\left(x\right)=\frac{a}{2}\left(\tanh\left(b\left(x-1\right)\right)+1\right)$$

In Equation 1, *a* represents the maximum response, the β-galactosidase activity in the experiments (*a*=85 Miller units for ethanol stress, and *a*=60 Miller units for NaCl stress) and RsbS phosphorylation in the simulation (*a*=0.2 for ‘product activation’). Parameter *b* encodes the sigmoidality, *i.e.* how fast the system switches between on and off (*b*=8*10^-1^/6*10^-3^ for ethanol/NaCl and *b*=12 for ‘product activation’). Parameter *c* encodes the inflection point; in the experiments this is the concentration of stressor producing half maximal β-galactosidase activity (*c*=3% for ethanol, and *c*=488 mM for NaCl), in the simulation this is the RsbR phosphorylation probability resulting in half maximal RsbS level (*c*=0.14 for ‘product activation’). The response (β-galactosidase and RsbS fractional phosphorylation) were divided by their associated estimated *a*-parameter in the hyperbolic tangent formula. For the experiment both signals, *i.e.* NaCl and ethanol concentrations, were divided by their respective *c* parameter. For the simulation the signal parameter, *kphr* (equivalent to RsbR phosphorylation), was divided by its associated *c* parameter. Thus, all data in the response range from zero to approximately one, and the response of 0.5, correlates to signal strength 1.

## Abbreviations

*B. subtilis*: *Bacillus subtilis*; UV light: Ultra-violet light; cryo-EM: Cryo-electromicroscopy; Rsb: Regulator of σ^B^; ADP: Adenosinediphosphate; ATP: Adenosinetriphosphate.

## Competing interests

The authors declare no conflict of interest.

## Authors’ contributions

UWL initiated the concepts for the study, performed modelling and simulation, and analysed the data. TM participated in the modelling and simulation, JMW, RJL and OW contributed to the conception and analysis. All authors edited, read, and approved the final manuscript.

## Supplementary Material

Additional file 1 **Figure S1.** Akbar et al. (2001) [13] (Figure 5A) studied beta-galactosidase expression for stressosomes composed only of RsbRC and RsbRD, of both of them (RsbRC+RsbRD) as well as a stressosome with all RsbR proteins (A+B+C+D). Although stressosome activation in the experiments (left) took place by transition to the stationary phase. RsbRC and RsbRD have been shown to be sensitive towards energy stress in *B. subtilis* (Martinez *et al*., 2010) [30]. The simplest way to reproduce the results of Akbar et al. (2001) [13], is to increase the phosphorylation parameter of RsbS, *kphs*. The open circles represent the wildtype with all *kphs* are RsbD stressosomes (Akbar et al. (2001) [13] left), reproduced in the simulation with an increase of *kphs* to 0.75 (filled circles, right). Akbar et al. (2001) [13] measured the highest background and stimulated response for a stressosome composed completely of RsbRC (filled squares, left). In the simulation a further increase in the response could be generated by an additional increase in kphs to 0.9 (tilled square, right). The experiments show that RsbRC and RsbRD are sensitive to energy stress but mixture with RsbRA and RsbRB lowers the overall stressosome sensitivity. In the simulation this is represented by a reduction of the RsbS phosphorylation rate. Click here for file

Additional file 2 **The Additional file 2 contains the matlab files for reproduction of the results.** To use it unpack all files into one directory. The main file is called liebal_stressosome-model_12_workflow-matlab.m. it is written using cell-mode. The cells can be evaluated sequentially by setting the cursor in a cell and evaluating it, eg. by Ctrl+Enter. Please contact ulfliebal@gmail.com for any questions. Click here for file
