# Supplementary material for: Simulations of stressosome activation emphasize allosteric interactions between RsbR and RsbT
Source: BMC Syst Biol. 2013 Jan 15;7:3. doi: 10.1186/1752-0509-7-3 (PMC3556497; doi:10.1186/1752-0509-7-3)
Supplement: Additional file 1 — Figure S1. Akbar et al. (2001) [13] (Figure 5A) studied beta-galactosidase expression for stressosomes composed only of RsbRC and RsbRD, of both of them (RsbRC+RsbRD) as well as a stressosome with all RsbR proteins (A+B+C+D). Although stressosome activation in the experiments (left) took place by transition to the stationary phase. RsbRC and RsbRD have been shown to be sensitive towards energy stress in B. subtilis (Martinez et al., 2010) [30]. The simplest way to reproduce the results of Akbar et al. (2001) [13], is to increase the phosphorylation parameter of RsbS, kphs. The open circles represent the wildtype with all kphs are RsbD stressosomes (Akbar et al. (2001) [13] left), reproduced in the simulation with an increase of kphs to 0.75 (filled circles, right). Akbar et al. (2001) [13] measured the highest background and stimulated response for a stressosome composed completely of RsbRC (filled squares, left). In the simulation a further increase in the response could be generated by an additional increase in kphs to 0.9 (tilled square, right). The experiments show that RsbRC and RsbRD are sensitive to energy stress but mixture with RsbRA and RsbRB lowers the overall stressosome sensitivity. In the simulation this is represented by a reduction of the RsbS phosphorylation rate. [file 1752-0509-7-3-S1.pdf]

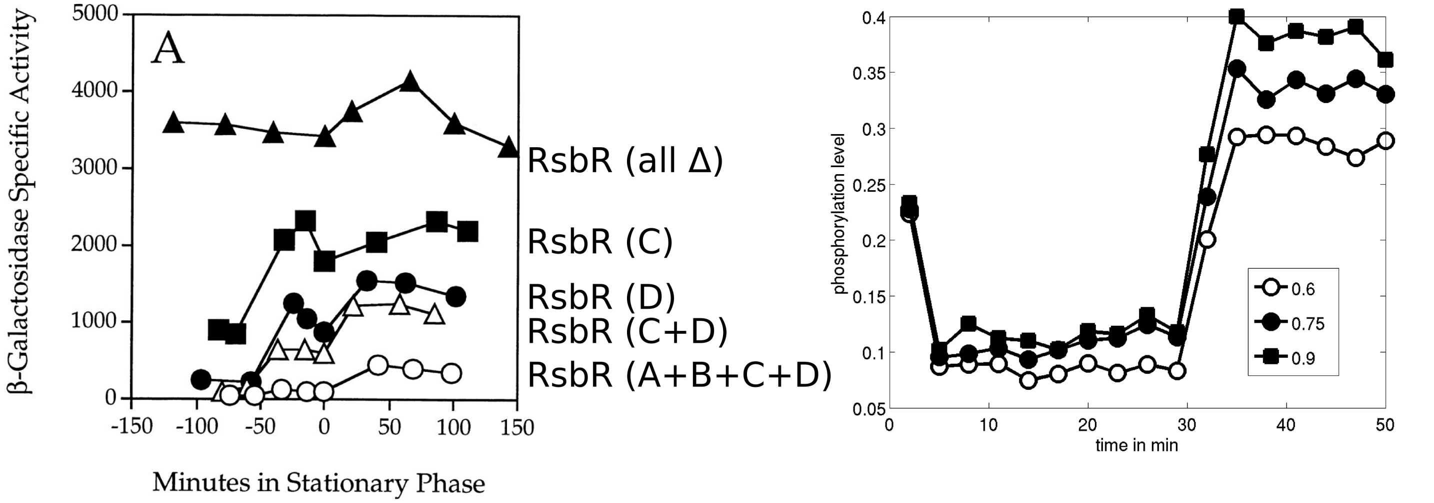

Supplementary Figure 1: Akbar et al. (2001) (Figure 5A) studied beta-galactosidase expression for stressosomes composed only of RsbRC and RsbRD, of both of them (RsbRC+RsbRD) as well as a stressosome with all RsbR proteins (A+B+C+D). Although stressosome activation in the experiments (left) took place by transition to the stationary phase, RsbRC and RsbRD have been shown to be sensitive towards energy stress in B. subtilis [Martinez et al., 2010, J Bacteriol]. The simplest way to reproduce the results of Akbar et al. (2001), to increase both the pre and post stimulus escitation, is to increase the phosphorylation parameter of RsbS, kphs. The open circles represent the wildtype with all four paralogues, that has a RsbS phosphorylation probability, kphs, of 0.6. The filled circles are RsbD stressosomes (Akbar et al. (2001) left), reproduced in the simulation with an increase of kphs to 0.75 (filled circles, right). Akbar et al. (2001) measured the highest background and stimulated response for a stressosome composed completely of RsbRC (filled squares, left). In the simulation a further increase in the response could be generated by an additional increase in kphs to 0.9 (filled square, right). The experiments show that RsbRC and RsbRD are sensitive to energy stress but mixture with RsbRA and RsbRB lowers the overall stressosome sensitivity. In the simulation this is represented by a reduction of the RsbS phopshorylation rate.
